# Fact-based nutrition for infants and lactating mothers—The NUTRISHIELD study

**DOI:** 10.3389/fped.2023.1130179

**Published:** 2023-04-18

**Authors:** Victoria Ramos-Garcia, Isabel Ten-Doménech, Alba Moreno-Giménez, Laura Campos-Berga, Anna Parra-Llorca, Amparo Ramón-Beltrán, María J. Vaya, Fady Mohareb, Corentin Molitor, Paulo Refinetti, Andrei Silva, Luis A. Rodrigues, Serge Rezzi, Andrew C. C. Hodgson, Stéphane Canarelli, Eirini Bathrellou, Eirini Mamalaki, Melina Karipidou, Dimitrios Poulimeneas, Mary Yannakoulia, Christopher K. Akhgar, Andreas Schwaighofer, Bernhard Lendl, Jennifer Karrer, Davide Migliorelli, Silvia Generelli, María Gormaz, Miltiadis Vasileiadis, Julia Kuligowski, Máximo Vento

**Affiliations:** ^1^ Neonatal Research Group, Health Research Institute La Fe (IISLAFE), Valencia, Spain; ^2^ Division of Neonatology, University & Polytechnic Hospital La Fe (HULAFE), Valencia, Spain; ^3^ Blood Transfusion Center from the Valencian Community, Valencia, Spain; ^4^ The Bioinformatics Group, Cranfield Soil and Agrifood Institute, Cranfield University, Bedford, United Kingdom; ^5^ REM Analytics S.A., Monthey, Switzerland; ^6^ Swiss Nutrition and Health Foundation, Epalinges, Switzerland; ^7^ Department of Nutrition and Dietetics, Harokopio University of Athens, Athens, Greece; ^8^ Research Division of Environmental Analytics, Process Analytics and Sensors, Institute of Chemical Technologies and Analytics, Technische Universität Wien, Vienna, Austria; ^9^ Quantared Technologies GmbH, Vienna, Austria; ^10^ Swiss Center for Electronics and Microtechnology (CSEM), Landquart, Suiza; ^11^ ALPES Lasers S.A., St Blaise, Switzerland

**Keywords:** preterm infants, lactation, human milk, breastfeeding, nutrition, donor human milk

## Abstract

**Background:**

Human milk (HM) is the ideal source of nutrients for infants. Its composition is highly variable according to the infant's needs. When not enough own mother's milk (OMM) is available, the administration of pasteurized donor human milk (DHM) is considered a suitable alternative for preterm infants. This study protocol describes the NUTRISHIELD clinical study. The main objective of this study is to compare the % weight gain/month in preterm and term infants exclusively receiving either OMM or DHM. Other secondary aims comprise the evaluation of the influence of diet, lifestyle habits, psychological stress, and pasteurization on the milk composition, and how it modulates infant's growth, health, and development.

**Methods and design:**

NUTRISHIELD is a prospective mother-infant birth cohort in the Spanish-Mediterranean area including three groups: preterm infants <32 weeks of gestation (i) exclusively receiving (i.e., >80% of total intake) OMM, and (ii) exclusively receiving DHM, and (iii) term infants exclusively receiving OMM, as well as their mothers. Biological samples and nutritional, clinical, and anthropometric characteristics are collected at six time points covering the period from birth and until six months of infant's age. The genotype, metabolome, and microbiota as well as the HM composition are characterized. Portable sensor prototypes for the analysis of HM and urine are benchmarked. Additionally, maternal psychosocial status is measured at the beginning of the study and at month six. Mother-infant postpartum bonding and parental stress are also examined. At six months, infant neurodevelopment scales are applied. Mother's concerns and attitudes to breastfeeding are registered through a specific questionnaire.

**Discussion:**

NUTRISHIELD provides an in-depth longitudinal study of the mother-infant-microbiota triad combining multiple biological matrices, newly developed analytical methods, and *ad-hoc* designed sensor prototypes with a wide range of clinical outcome measures. Data obtained from this study will be used to train a machine-learning algorithm for providing dietary advice to lactating mothers and will be implemented in a user-friendly platform based on a combination of user-provided information and biomarker analysis. A better understanding of the factors affecting milk's composition, together with the health implications for infants plays an important role in developing improved strategies of nutraceutical management in infant care.

**Clinical trial registration:**

https://register.clinicaltrials.gov, identifier: NCT05646940.

## 1. Introduction

Breastfeeding (BF) is the optimal feeding practice for all infants, associated with numerous health benefits for the mother–infant dyad, as well as, being an ecologic practice (1). The World Health Organization highly recommends exclusive BF for all infants up to six months, born either full–term (2, 3) or prematurely (4). Human Milk (HM) is a dynamic fluid that meets the offspring's needs (5–8), and is ample in nutrients and bio-active compounds intended to enhance immunity and growth (9). HM also hosts a complex ecosystem of microbiota (10), which is of paramount importance for the offspring's immunity (5).

During the last decades, the incidence of preterm deliveries (<37 weeks of gestation) and survival rate of preterm infants (PI) have been steadily increasing (11), hand in hand with the interest in preterm and early infant nutrition. Associated birth complications are the leading cause of death among children under five years of age, responsible for ∼1 million global deaths per year (12). Progress in medical interventions has allowed to enhance survival of an increasing proportion of extremely low gestational age newborns and low birth-weight infants. Early infant nutrition has become a major player in improving clinical outcomes of survivors (13). HM is recommended for PI based on an impressive array of benefits provided to this highly vulnerable population, including ameliorated immunological and gastrointestinal outcomes (14–20). Optimal growth in the postnatal period is challenging dure to increased nutritional demands and metabolic and digestive immaturity of PI. Greater weight gain is associated with better neurodevelopment outcomes (21).

In situations where mothers are unable to produce sufficient milk quantities to exclusively or partially breastfeed, pasteurized donor human milk (DHM) is a viable option to avoid formula feeding, especially for low birth-weight children (22). DHM is a valuable but limited resource and Human Milk Banks prioritize its distribution to the patients with highest risk of necrotizing enterocolitis, generally PI <32 weeks of gestation or with a birthweight <1,500 g (3, 23). To date, most studies focus on the benefits of using DHM over formula in PI, when own mother's milk (OMM) is limited or unavailable. There is clear evidence of the superiority of OMM against formula (24); however, although DHM apparently protects against necrotizing enterocolitis as compared to formula, results are not conclusive (25) and there are only few reports directly comparing DHM and OMM. While providing some bioactive agents, DHM consumption is associated with slower growth rates in comparison to the administration of OMM or formula (24). This finding may be attributable to several factors. Most DHM is provided by women who have delivered at term and donate their milk in later stages of lactation up to several months after delivery. The effect of gestational age on HM composition has been reported to affect a wide array of compounds including lipids (26), lactoferrin (27), amino acids (28), and HM oligosaccharides (29). In comparison to preterm milk during the first weeks after delivery, studies on the mean composition of DHM show a lower content in total protein, fat, and other bioactive molecules (30). Its composition is also affected by the processing of expressed milk, including stringent protocols applied in HM banks, i.e., pasteurization, freezing, and storage (31, 32), necessary to reduce the potential risk to transmit infectious agents.

Beyond the physiological adaptations, research has focused on the potential effects of the maternal diet on the HM composition. In an earlier report, HM composition was found rather different among mothers of diverse ethnic backgrounds, with different dietary habits (33). The fatty acid profile of HM varies in relation to maternal diet, particularly, in the long chain polyunsaturated fatty acids (LCPUFAs) (31, 32, 34, 35). Additionally, in a recent comprehensive systematic review of 104 observational and interventional studies, HM composition was found to relate to the maternal consumption of fatty acids, fat-soluble vitamins, and vitamin B1 and C intake (36, 37). No similar relationships were found for dietary intake of iron, folate, calcium, selenium, or proteins (36).On the contrary, another recent systematic review determined that information regarding the relationship between dietary patterns during lactation and HM content is insufficient for total fat, vitamins B and C, and choline content and inexistent for total proteins, macronutrient distribution, human milk oligosaccharides (HMOs), vitamins A, D, E and K, iodine, and selenium (38). Hence, more studies are needed to conclude for several nutrients and little is known on how the maternal diet may also affect non-nutritive constituents of HM, e.g., prebiotics content (39) or microbiota (40, 41). Finally, existing evidence comes mainly from studies on full-term infants (TI); the impact of maternal nutrition on the composition of HM for the preterm offspring has not been adequately documented.

Therefore, the main objective of this study is to compare the % weight gain/month in PI and TI exclusively receiving either OMM or DHM. In addition, secondary objectives are (i) to evaluate associations between the mother's diet, physical, and psychosocial status (e.g., lifestyle, perceived stress, anxiety, and depression) and HM composition in PI and TI, (ii) to evaluate the effect of pasteurization/storage on DHM composition, (iii) to assess the interplay of microbiota and microbiota activity and HM composition, (iv) to assess the interplay of microbiota and microbiota activity detected in HM and PIs and TIs, (v) to evaluate the impact of microbiota and microbiota activity on the vitamin status of PIs and TIs, (vi) to test the performance of novel sensor devices developed within this project, and (vii) the development of a personalized nutrition algorithm for lactating mothers.

## 2. Methods and analysis

### 2.1. Study design

The NUTRISHIELD study is a parallel group, non-randomized, observational study performed at the Division of Neonatology of the University & Polytechnic Hospital La Fe (HULAFE), including TI and PI and their mothers, covering the period from birth to 24 months of infant's age. In addition, mothers providing DHM to study participants are also included.

The study protocol has been approved by the Scientific and Ethics Committee for Biomedical Research (CEIm) of the HULAFE (#2019-289-1). All methods have been performed in accordance with the relevant guidelines and regulations and written permission has been obtained from mothers or legal representatives by signing an informed consent form.

Confidentiality of subjects is maintained during the study. All participants have been assigned a code and data allowing personal identification will not be shared at any time. Participants may withdraw consent for participating in the study at any time.

### 2.2. Study population and recruitment

Participants were recruited at the end of pregnancy (when admitted to the obstetric ward) or within one week after birth in case of hospitalized infants at HULAFE. Recruitment started in October 2020 and was completed in July 2022. Three mother-infant groups were enrolled, i.e., PI fed with OMM (PI-OMM), PI fed with pasteurized DHM (PI-DHM), and TI receiving OMM (TI-OMM), and their mothers.

The inclusion criteria were (i) acceptance of the mother to participate and sign an informed consent form, (ii) a gestational age <32 weeks for the group of PI and >37 weeks for the group of TI, and (iii) exclusive consumption (i.e., >80% of total intake) of either OMM or DHM at time point CEN (complete enteral nutrition, 150 mL/Kg/day) for PI or RBW (recovery of birth weight) for TI. Although the type of feeding of PIs included in either group (PI-OMM or PI-DHM) may have changed at later time points, they remained in the same study group, so the effect of feeding of each type of milk at CEN and its long-term effects can be studied. Likewise, the use of infant formula and the initiation of weaning at later time points was recorded without affecting follow-up assessments. The exclusion criteria were (i) non-compliance with any of the inclusion criteria, (ii) the requirement of a special diet for the mother (e.g., celiac disease, diabetes) or maternal consumption of probiotics, (iii) the need of intestinal surgery, severe congenital malformations, or chromosomopathies of the infant, (iv) mother's residence outside the Valencian Community and (v) severe language barriers hampering the collection of necessary data from mothers not speaking Spanish and/or English.

Sample size estimation was performed for the primary outcome (i.e., % weight gain/month from birth until hospital discharge of PI fed with DHM and OMM) and was based on result of a previous study (42). Considering the median % weight gain/month (interquartile range, IQR) of 52% (IQR: 30) in the PI-DHM group and 63% (IQR: 21) in the PI-OMM, 18 infants per group were needed to achieve a power of 90% with an alpha error of 5% between the % weight gain/month of PI exclusively receiving either OMM or DHM at CEN.

On the other hand, HM donors were recruited during their regular visits at the hospital's HM Bank of the Valencian Community. All participants met ordinary criteria for HM donation (e.g., negative screening results of a series of transmissible diseases, toxic habits, or some chronic medication) and accepted to sign an informed consent form and participate in the study.

### 2.3. Assessment points and biological samples

**Figure 1:**
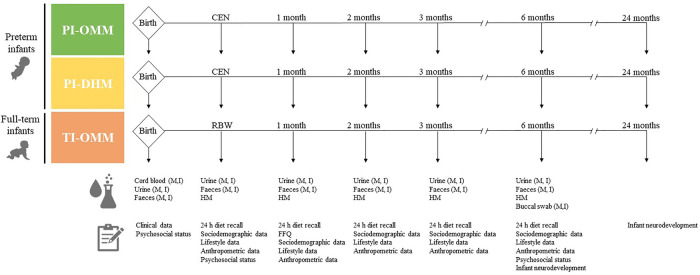
Design of the NUTRISHIELD study. PI, preterm infant; TI, term infant; M, mother; I, infant; OMM, own mother's milk; DHM, donor human milk; CEN, complete enteral nutrition (150 ml/kg/day); RBW, recovery of birth weight; HM, human milk; FFQ, food frequency questionnaire.

Samples from lactating mothers and their infants are collected at different timepoints, as shown in Figure 1. At birth, cord blood, and urine, and faeces from both infants and mothers are collected when possible. When PIs achieve CEN and TIs RBW, as well as one, two, three, and six months after delivery, urine and faeces from mothers and infants, as well as, HM from lactating mothers, are collected. Infants' and mothers' buccal swabs are collected at month six. During hospitalization of participants, samples are collected at the hospital by healthcare providers. After discharge, samples are collected at home and either transported to the hospital by study participants or staff of the NUTRISHIELD study. Mothers receive Standard Operation Procedures (SOPs) with detailed instructions and all the required material for sample collection at home. Additionally, hospital's staff instructs mothers on how to collect and store samples correctly.

Samples collected at home are stored at 4°C during a maximum of 24 h before their transport to the hospital on ice, where they are registered, aliquoted, labelled, and stored at −80°C until analysis. A HM and faeces aliquot is directly stored in a tube containing a microbiome preservative solution. For DHM, collection times respective to delivery are heterogeneous, since from each donor several aliquots are collected over time, pooled, and then subjected to Holder pasteurization in batches (i.e., 62.5°C during 30 min followed by rapid cooling to 4°C). The lactation time was estimated as the mean (standard deviation, SD) of time elapsed during the collection of different aliquots from one DHM batch, being 16 (SD 12) days.

**Figure 2:**
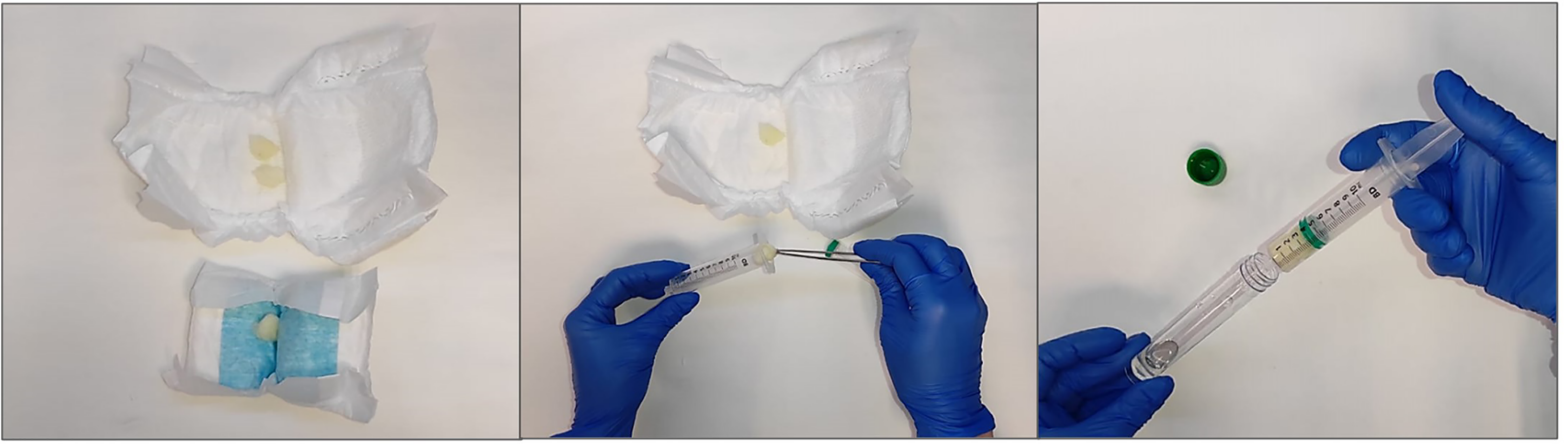
Infants’ urine sample collection procedure. Sterile cotton pads are placed in the diaper (left); cotton pads soaked with urine are collected using sterile tweezers (middle); cotton pads are squeezed with a sterile syringe to collect the urine sample (right).

Mothers' and infants' buccal swab samples are collected by thoroughly rubbing a flocked sterile swab up and down the inner side of both cheeks for 30 s each. Swabs are cut and stored in a sterile tube at −80°C until further analysis. Discarded arterial and venous blood from the umbilical cord is collected in EDTA blood sample tubes after the placenta is delivered and separated from the baby. Blood is centrifuged (1,300 × *g* for 10 min at 20°C) and the upper plasma layer is stored in opaque vials at −80°C until analysis. Mother's first morning urine is collected in a polypropylene container, and infant's urine is collected by placing sterile cotton pads in the diaper and, after urinating, squeezing them with a sterile plastic syringe into sterile tubes (see Figure 2). Mother's faeces are collected in a polypropylene container, and infant's faeces are collected directly from the diaper into sterile tubes using sterile tweezers.

Mothers express milk using breast milk pumps following an SOP employed routinely in the hospital and the HM bank. The removable parts of the breast milk pump as well as the collection bottles are sterilized before their use. HM is collected at least three hours after breastfeeding. Mothers are required to wash their hands with soap and water and clean nipples with water. HM should be preferably expressed between 7 and 10 a.m., full expression of one breast is required (a minimum volume of 50 ml is recommended), and details regarding the extraction method (e.g., breast pump brand), date, and extracted volume are registered. For DHM samples, aliquots of each pooled DHM sample are collected before and after Holder pasteurization.

### 2.4. Dietary and psychological status assessments

For all three study groups, information obtained from clinical records is collected. Anthropometric data (e.g., weight, height, and head circumference), sociodemographic, dietary, other relevant information related to lifestyle (e.g., smoking habits, sleep hours, and physical activity), and psychosocial status are provided through questionnaires by participating mothers as summarized in Table 1. As shown in Figure 1, 24 h dietary recalls are recorded at all timepoints except at birth, food frequency questionnaire (FFQ) at month 1, maternal psychosocial status at CEN/RBW and month six (corrected age for PI groups), and infant neurodevelopment at months six and twenty-four. The questionnaires are administered online, on paper, or through direct interviews with the mothers.

**Table 1:** Recorded parameters from lactating mothers and infants.

Assessment	Parameter
Nutrition	FFQ
	24-hour dietary recall
Clinical data	Maternal conditions
	Pregnancy complications
	Delivery type
	Medical information
	Maternal medication
	IIFAS
	Infant's gestational age
	Infant sex
	Infant diagnosis
	Perinatal complications
Sociodemographic data	Origin
	Education
	Household income
	Employment status
Lifestyle	Smoking habits
	Physical activity
	Sleeping habits
Anthropometric data	Maternal weight
	Infant height
	Infant head circumference
Psychosocial status	MSPSS
	FACES
	PSS-10
	STAI
	EPDS
	BDI
	TEQ
	PBQ
	PSI
Infant neurodevelopment	ASQ-3
	IBQ
	Merril-Palmer revised scales

FFQ, Food Frequency Questionnaire; IIFAS, Iowa Infant Feeding Attitude Scale; BMI, Body Mass Index; MSPSS, Multidimensional Scale of Perceived Social Support; FACES, Family Adaptability and Cohesion Evaluation Scale; PSS-10, Perceived Stress Scale-10; STAI, State-Trait Anxiety Inventory; EPDS, Edinburgh Postnatal Depression Scale; BDI, Beck Depression Inventory; TEQ, Traumatic Experience Questionnaire; PBQ, Postpartum Bonding Questionnaire; PSI, Parenting Stress Index; ASQ-3, Ages & Stages Questionnaires; IBQ, Infant Rothbart's Temperament Questionnaire.

Regarding the 24 h recall, trained researchers ask for all foods and beverages participants consumed during the previous day, using the multiple-pass method (43). Recall data are analyzed in terms of nutrients using the dietary analysis software Nutritionist Pro™ (2007, Axxya Systems, Texas, USA). Additionally, dietary intake is grouped into food groups, (i.e., fruits, vegetables, bread/starch, meat/high fat, meat/medium fat, meat/low fat, meat/very low fat, milk/non-fat, milk/low fat, milk/full fat, eggs, fish, soy, dairy products, soft drinks, and other carbohydrate-rich foods).

The FFQ is administered by trained personnel, and it comprises 142 questions on the consumption of foods that are commonly eaten by the Spanish population throughout a year, including dairy products, cereals, fruits, vegetables, meat, fish, legumes, added fats, alcoholic beverages, stimulants, and sweets. Using a 9-grade scale (“never or less than 1 time/month”, “1–3 times/month”, “1 time/week”, “3–4 times/week”, “5–6 times/week”, “1 time/day”, “2–3 times/day”, “4–5 times/day”, “≥6 times/day”) participants are required to indicate the absolute frequency of consuming a certain amount of food, expressed in grams, milliliters or in other common measures, such as slice, tablespoon, or cup, depending on the food. The previous month is set as the timeframe. The FFQ is an easy-to-use questionnaire and is not expected to increase the burden of lactating mothers.

Based on the FFQ-responses, adherence to the Mediterranean diet is evaluated by using the MedDietScore, a composite score calculated for each participant (44). For food groups presumed to be part of the Mediterranean pattern (i.e., those with a recommended intake of 4 servings per week or more, such as non-refined cereals, fruits, vegetables, legumes, olive oil, fish, and potatoes) higher scores are assigned when the consumption is according to the rationale of the Mediterranean pattern, while lower scores are assigned when participants report no, rare, or moderate consumption. For the consumption of foods presumed to be eaten less frequently within the Mediterranean diet (i.e., consumption of meat and meat products, poultry, and full fat dairy products), scores are assigned on a reverse scale. As this study is focused on lactating mothers, the original score was modified by removing the alcohol consumption component. Thus, the range of this modified MedDietScore is between 0 and 50, with higher values of the score indicating greater adherence to the Mediterranean diet.

Maternal psychosocial status is measured at recruitment and at six months, including social support [Multidimensional Scale of Perceived Social Support, MSPSS (45)], family functioning [Family Adaptability and Cohesion Evaluation Scale, FACES (46)], perceived stress [Perceived Stress Scale -10, PSS-10 (47)], anxiety [State-Trait Anxiety Inventory, STAI (48)], depression [Edinburgh Postnatal Depression Scale, EPDS (49), Beck Depression Inventory (BDI) (50)] symptoms, and traumatic life events [Traumatic Experience Questionnaire, TEQ (51, 52)]. Mother-infant postpartum bonding [Postpartum Bonding Questionnaire, PBQ (53)] and parental stress [Parenting Stress Index, PSI (54)] are also examined.

As for the infants, at six and twenty-four months (corrected age for PIs) assessment of neurodevelopment is conducted during a hospital visit by a trained psychologist and psychiatrist from the team. Parents complete the Ages & Stages Questionnaires, Third Edition (ASQ-3) (55) for psychomotor development evaluation and the Infant Rothbart's Temperament Questionnaire (IBQ) (56) for infant temperament. The ASQ is a well standardized instrument used in clinical and research practice to examine children's psychomotor development gathering items in five domains: communication, gross motor, fine motor, problem solving, and personal-social. Clinical observation and Merrill-Palmer revised scales (57) for development are applied during the session.

Mother's concerns and attitudes to breastfeeding are also registered through the Iowa Infant Feeding Attitude Scale (IIFAS) (58) at CEN/RBW and six months.

Milk donors are asked to provide sociodemographic, medical, dietary, and other lifestyle data. PSS-10 is evaluated when entering the study in order to correlate with hormones and other compounds present in the milk. In particular, each time a batch of milk is prepared, a FFQ is administered (representing the period of time that the batch of milk covered) and the donors are asked to provide medical, anthropometric, and lifestyle data.

### 2.5. Analysis of biological samples employing laboratory methods

A range of state-of-the-art as well as novel laboratory methods are employed for the analysis of the collected biological samples, as summarized in Table 2.

**Table 2:** Analysis of biological samples employed.

Analysis	Parameters	Technique	Matrix
Genome	Whole genome sequencing	Illumina NovaSeq	Buccal swab
Microbiota	Bb species	ATGC	Faeces
			HM
Microbiota activity	SCFAs BCAAs	GC-MS	Faeces
			Urine
			HM
	Bas	LC-MS/MS	Urine
Nutrition	Flavonoids	LC-MS/MS	Urine
	Isoflavones		
	Arylglycines		
	Amino acids		
Metabolome	Metabolomic fingerprinting	LC-HRMS	Urine
Macronutrients	Fat	Miris HM analyzer	HM
	Carbohydrates		
	Crude & true protein		
	Total solids		
	Energy		
Fatty acids	34 fatty acids (C6-C24)	GC-MS	HM
	SAT	HM fatty acid sensor	
	MONOs		
	PUFAs		
	UNSAT		
	SCFAs		
	MCFAs		
	LCFAs		
Vitamins	Vitamin B, D and K groups	LC-MS/MS	HM Umbilical cord blood
	Retinol forms	LC-UV	
	Carotenoids and vitamin E groups	LC-UV/Fluorescence	
HMOs	HMO screening	LC-HRMS	HM
Steroids	19 steroid hormones	LC-MS/MS	HM
			Urine
Proteins	Total protein	HM protein sensor	HM
	Alpha-lactalbumin		
	Lactoferrin		
	Casein		
pH	pH	Urine pH sensor	Urine
Phosphate and creatinine	Phosphate and creatinine	Phosphate and creatinine sensor	Urine

ATGC, Advanced Testing for Genetic Composition; Bb, Bifidobacterium; GC, Gas Chromatography; (HR)MS, (High Resolution) Mass Spectrometry; SCFAs, Short Chain Fatty Acids; BCAAs, Branched Chain Amino Acids; Bas, Bile Acids; SAT, saturated fatty acids; MONOs, monounsaturated fatty acids; PUFAs, polyunsaturated fatty acids; UNSAT, unsaturated fatty acids; MCFAs, medium-chain fatty acids; LCFAs, long-chain fatty acids; LC, Liquid Chromatography; UV, Ultraviolet; HMOs, Human Milk Oligosaccharides.

#### 2.5.1. Genome sequencing

For genome sequencing analysis, DNA extraction from buccal swab samples takes place according to the protocol developed by the sequencing provider facility. Polymerase chain reaction (PCR) quality control (QC) is performed before samples are shipped to the sequencing service provider, and again upon receival by the sequencing facility. QC and library preparation follow the Illumina NovaSeq protocol (Paired End, 150 bp). Three polygenic risk scores (PRS) models are developed for body mass index (BMI), diabetes type 2, and lactose intolerance, calculated by Plink software (59), and the resulting genotype files, one per sample, are then used as separate input to the PRS models.

This score can then be compared to the scores obtained from the UK Biobank set (60), to estimate the relative risk for this individual compared to the rest of the UK Biobank individuals (∼370.000 individuals), which are split in 10 quantiles according to their genetic risk.

#### 2.5.2. Microbiota analysis

Advanced Testing for Genetic Composition (ATGC) is used for microbiota analysis of faeces and HM samples (61). It is a targeted measurement technique that provides greater precision, specificity, and versatility than rtPCR, while being just as cost-effective and fast. It has been developed to translate discovery data from untargeted analysis (e.g., 16S or shotgun meta-genomics) into tests for routine use.

The Bifidobacterium (Bb) assay used is specifically designed to analyze the main species and subspecies varieties in HM and newborn gut microbiomes. For sample processing, the HM lipid layer is solubilized using a detergent, while for faeces, a lysis step using a lysis buffer with bead beating for 20 s is required. Then, an in-house DNA extraction procedure and a PCR are carried out. Cycling temperature capillary electrophoresis (CTCE) is made for each primer on an independent capillary. It is performed on a MegaBace 1,000 instrument (General Electrics, Boston MA, USA) as described earlier (62).

#### 2.5.3. Microbiota activity biomarkers analysis

The microbiota activity is evaluated with the following methods:

(i) Short chain fatty acids (SCFAs) and branched chain amino acids (BCAAs) in faeces, urine and HM samples

SCFAs (i.e., acetic, propionic, isobutyric, butyric, 2-methylbutyric, isovaleric, valeric, caproic and heptanoic acids) and BCAAs (i.e., valine, leucine and isoleucine) are determined by targeted gas chromatography coupled to mass spectrometry (GC-MS), as described elsewhere (63, 64), using an Agilent 7890B GC system coupled to an Agilent 5977A quadrupole MS detector (Agilent Technologies, Santa Clara, CA, USA).

(ii) Bile acids (BAs) in urine samples

In total, 34 BAs (6 primary, 7 primary conjugated, 5 secondary, 10 secondary conjugated and 6 sulphated) are determined in urine samples by liquid chromatography coupled to tandem MS (LC-MS/MS), as described elsewhere (65) with minor modifications, using an ACQUITY LC chromatograph (Waters Ltd, Elstree, UK) coupled to a Xevo TQ-S MS detector (Waters, Manchester, UK).

#### 2.5.4. Nutrition biomarkers analysis

Quantification and semi-quantification of 20 and 205 urinary nutrition biomarkers (e.g., flavonoids, isoflavones, and arylglycines), respectively, related to nine food groups such as fruits, vegetables, meat, fish, dairy products, milk, seeds, coffee, and soft drinks is carried out (66, 67). Additionally, seven microbiota activity biomarkers are determined using this method (i.e., phenylpropionylglycine, L-kynurenine, L-tyrosine, hippuric acid, 3-indolepropionic acid, ferulic acid sulphate and 3-indoleacetic acid). LC-MS/MS analysis is conducted using a Sciex QTRAP 6500 + system (Sciex, Framingham, Massachusetts, USA).

#### 2.5.5. Untargeted metabolomic fingerprinting

Untargeted metabolomic analysis is carried out in urine samples by LC-MS/MS, employing an Agilent 1,290 Infinity HPLC system coupled to an Agilent 6,550 Spectrometer iFunnel quadrupole time-of-flight (QTOF) MS detector (Agilent Technologies, Santa Clara, CA, USA), as previously described (68).

#### 2.5.6. Macronutrients analysis

Direct measurement of HM macronutrients including fat, carbohydrates, crude and true proteins, total solids, and energy are determined using a Miris HM analyzer (Miris AB, Uppsala, Sweden). Determinations, QC, and instrument calibration are performed following the SOP provided by the manufacturer (69).

#### 2.5.7. Fatty acid profile analysis

The targeted analysis of 36 fatty acids in HM samples is carried out by GC-MS as described elsewhere (70, 71), using an Agilent 7890B GC system coupled to an Agilent 5977A quadrupole MS detector (Agilent Technologies, Santa Clara, CA, USA).

#### 2.5.8. Vitamin analysis

Quantification of water-soluble (group of vitamin B: thiamine, thiamine monophosphate, riboflavin, flavin adenine dinucleotide, nicotinamide, pyridoxal and pyridoxal phosphate) and lipid-soluble vitamins (group of vitamin A: retinol forms, β-carotene, β -cryptoxanthin, lutein, lycopene and zeaxanthin; group of vitamin E: α-tocopherol and γ-tocopherol; group of vitamin K: phylloquinone and menaquinone-4; group of vitamin D: cholecalciferol and calcifediol) in HM and umbilical cord blood samples is carried out.

The analysis of B, D and K vitamins are determined as described somewhere (72–76), using an Acquity LC chromatographic system (Waters AG, Switzerland) hyphenated to a Xevo TQ-S mass spectrometer (Waters AG, Switzerland).

The retinol forms are analyzed as previously described (75), using a LC using a quaternary Flexar chromatographic system (Perkin Elmer, Switzerland) with UV detection (325 nm).

Carotenoids and vitamin E analysis is carried out as previously described (77), using a binary Acquity LC chromatographic system (Waters AG, Switzerland) with multiple UV detection (295 nm, 450 nm and 472 nm) and fluorescence detection (exc. 296 nm/em. 330 nm).

#### 2.5.9. Oligosaccharides analysis

HMOs analysis is carried out by LC-MS in accordance with a previously described protocol (78, 79) with some modifications, employing a Vanquish LC Binary Pump coupled to an Orbitrap QExactive Plus MS detector (ThermoFisher, Waltham, MA, USA).

#### 2.5.10. Steroid analysis

Steroid analysis of HM and urine samples is carried out on an Acquity UPLC system (Waters Ltd, Elstree, UK) coupled to a Waters Xevo TQ-S MS detector (Waters, Manchester, UK) as previously described (80, 81). A panel of 19 steroids is targeted (i.e., cortisol, 5β-tetrahydrocortisol, 6β-hydrocortisol, 20α-dihydrocortisol, 20β-dihydrocortisol, corticosterone, aldosterone, estrone, androstenedione, dehydroepiandrosterone, progesterone, 17-hydorxy-progesterone, pregnenolone, cortisone, 20α-dihydrocortisone, 20β-dihydrocortisone, 6α-hydroxycortisone, testosterone, and 5α-dihydrotestosterone).

### 2.6. Sensor prototypes

Due to the special requirements of the study, three sensor prototypes enabling the determination of complementary parameters in HM and urine samples are being developed and benchmarked within the NUTRISHIELD study.

#### 2.6.1. HM protein sensor

Direct quantification of the most abundant proteins (i.e., casein, *α*-lactalbumin, and lactoferrin) in HM milk is performed using quantum cascade laser-based mid-infrared (QCL-IR) spectroscopy (82, 83). Laser-based IR spectroscopy enables more robust and sensitive analysis in the spectral region of IR signatures of proteins compared to conventional Fourier-transform infrared (FTIR) spectrometers (82). Reference protein analysis is carried out using specific Kjeldahl and HPLC analysis. Within the study, a dedicated QCL-based protein analyzer for HM is developed and benchmarked.

#### 2.6.2. HM fatty acids sensor

HM fatty acid profiling is performed based on mid-IR spectroscopy of an extracted lipid HM fraction. For analysis, 15 µl of the pure fat are transferred onto a diamond single bounce attenuated total reflection (ATR) accessory (Platinum ATR, Bruker, Ettlingen) connected to a Tensor 37 (Bruker, Ettlingen) FTIR spectrometer (84, 85).

#### 2.6.3. Urine pH sensor

A custom-made portable system based on potentiometric measurements and screen-printed electrode technology is developed to measure pH in urine. The system works with small sample volumes (i.e., 50 μl) and no dilution is needed. An additive is spiked into samples prior to measurement in order to preserve and regenerate the sensor's surface, which extends sensor lifetime to multiple uses with no carry-over between urine samples.

#### 2.6.4. Urine phosphate and creatinine sensor

Quantification of phosphate and creatinine in urine samples is performed using a method based on FTIR transmission spectroscopy. Within the study, a dedicated QCL-based urine analyzer is developed. Reference values for phosphate are obtained by a colorimetric determination after reaction of inorganic phosphorus with ammonium molybdate, while creatinine reference values are obteined following the manufacturer's instructions of the modified Jaffe's method implemented in the DetectX® urinary creatinine detection kit from Arbor Assays (Ann Arbor, MI, USA).

### 2.7. Statistics and data integration

The Student's *t*-test, Wilcoxon ranksum test or *Χ*^2^ test with *α* = 0.05 will be used for between-group comparisons and fold changes (FC) will be calculated as the ratio of means or medians between groups in accordance to the underlying distribution of the data. Pearson's linear or Spearman rank correlation coefficients will be determined between continuous variables. If necessary, partial correlation coefficients adjusting for known confounding factors will be computed. The false discovery rate (FDR) from the *p*-values of multiple-hypothesis testing will be estimated using the Benjamini and Hochberg procedure (86) and adjusted *p*-values <0.05 will be considered statistically significant.

One of NUTRISHIELD secondary objectives is the development of a personalized nutrition system based on measured biomarkers. The system is to be made available to medical personnel, as well as mothers, to improve infant-mother dyads' health and wellbeing. The results and the associations revealed from the study will be used to train a personalized nutritional algorithm with data obtained from clinical settings. This will be used to build and validate the NUTRISHIELD platform, describing how a theoretical framework designed and fed by the patients' data translates to clinical practice. Toward that goal, a comprehensive data integration system has been developed as the Clinical Trial App (CTA). The CTA is a system intended for personalized medicine/nutrition, implementing the following main features: (i) data acquisition from questionnaires, (ii) data acquisition from laboratory biomarker analysis, (iii) processing data to train machine learning algorithms, and (iv) deploying trained algorithms to produce reports for final users.

**Figure 3:**
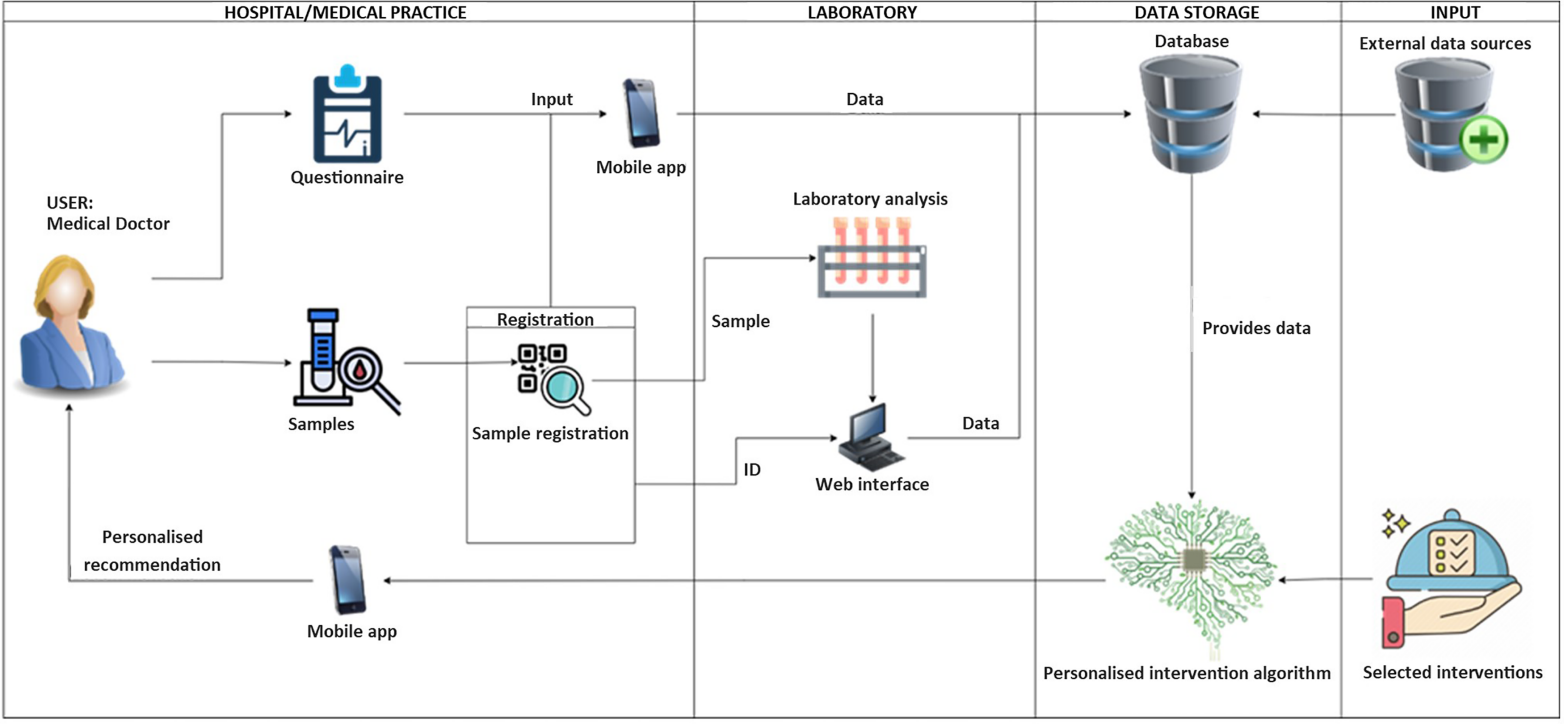
Data flow and functionalities of the Clinical Trial App (CTA).

Figure 3 shows the data flow and functionalities of the CTA. Questionnaires data are collected by medical staff directly using the phone app. Alternatively, questionnaires that have been collected on paper or on spreadsheets, can be uploaded as well. All samples can be identified with a unique QR code and, at the moment of collection, the phone app can be used to scan the QR code and input sample data. This way the samples can travel between labs with the QR code as only identification. The QR code can be scanned again to associate the results with the original sample when uploading it through the web portal. The collected data is stored on a database and used to feed the personalized recommendation algorithm. This algorithm produces a report and feeds it back to the phone app.

Using the CTA, all study data is consolidated in a structured database, of which an anonymized version can be later stored on public repositories for re-use in further research.

## 3. Results and discussion

**Figure 4:**
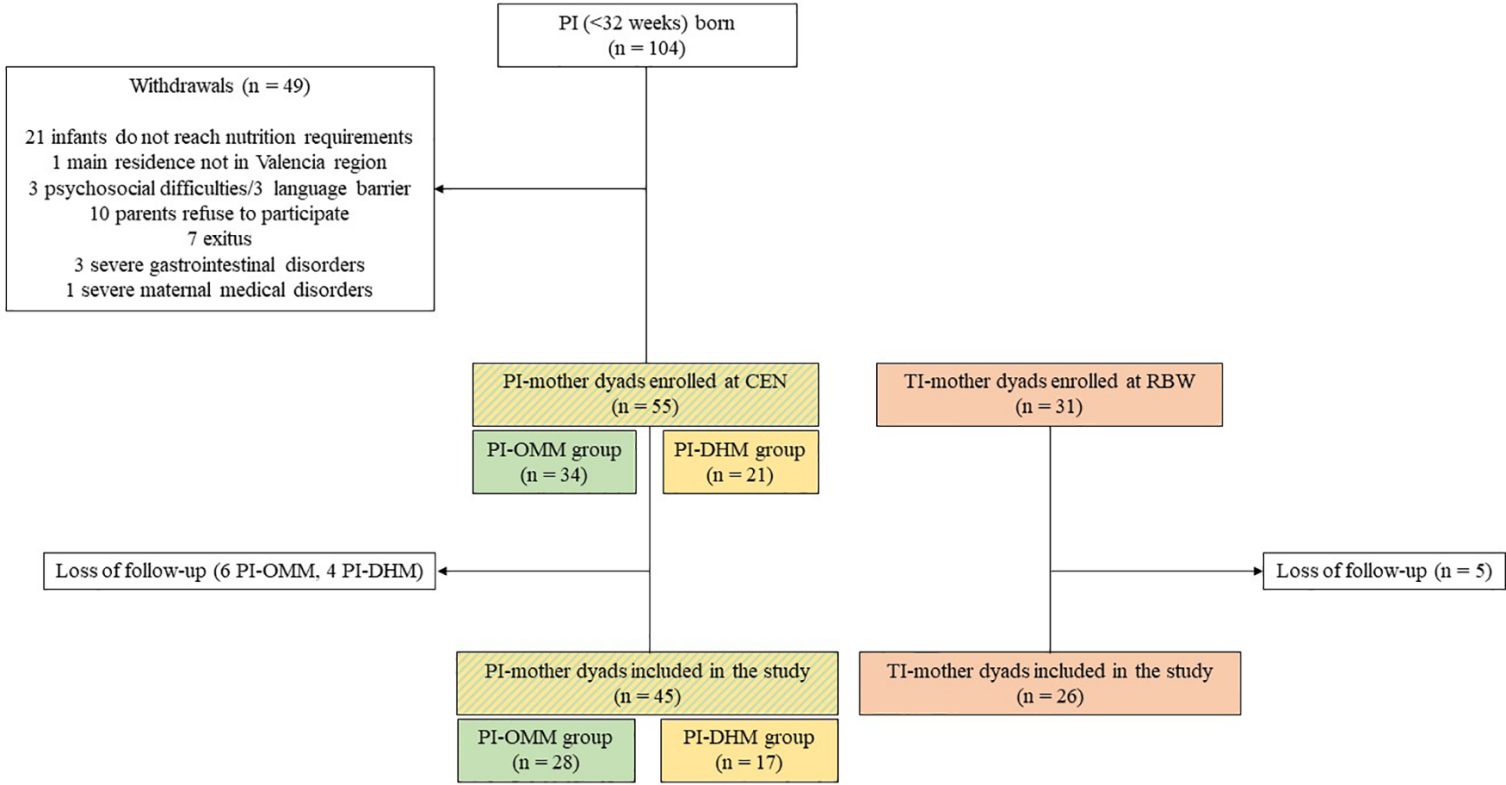
Prospective flow chart of the NUTRISHIELD study. PI, preterm infant; TI, term infant; CEN, complete enteral nutrition (150 ml/kg/day); RBW, recovery of birth weight; OMM, own mother's milk; DHM, donor human milk.

Figure 4 summarizes the recruitment process, including the eligible PI (*n* = 104), excluded (*n* = 49), lost to follow up (*n* = 10) and the number of PI that were finally included in the study (*n* = 45). In addition, a control group comprising 31 full term OMM infant-mother dyads was recruited, with five participants being lost during follow-up. Altogether, a total of 71 infant-mother dyads were included and completed the study (28 PI-OMM, 17 PI-DHM and 26 TI-OMM), after losing 15 infant participants during follow-up. In total 662 urine, 594 faeces, 134 buccal swab, 33 venous and arterial cord blood, and 234 HM samples, as well as aliquots from 147 DHM batches before and after pasteurization have been collected. An ample array of laboratory methods for biomarker analysis and screening of biological samples and three *ad-hoc* designed sensors were developed. Currently, the analysis of all collected study samples is in progress at the different participating institutions.

The results obtained from HM analysis will be used to study how macronutrients, proteins, vitamins, HMOs, steroids, fatty acids, and microbiota are affected by maternal nutrition, psychosocial status (e.g., social support, family functioning, and perceived stress), other clinical variables (e.g., gestational age, BMI, and type of delivery), and pasteurization, in the case of DHM. Additionally, it is assessed how HM composition affects growth and other health parameters in TI and PI. The results obtained from urine samples analysis will also be used to study how urinary nutrition biomarkers and the metabolic profile are affected by nutrition, psychosocial status, the PRS obtained from genome sequencing and other clinical variables. In addition, HM and faeces microbiome analysis will be used to study its correlation with microbiota biomarker analysis (i.e., SCFAs, BCAAs, and BAs). HM and urine samples will also be used to test the pH, protein, fatty acids, and phosphate and creatinine sensors developed within the study.

This study presents several strengths and limitations. During the study design, special emphasis was put on the comfort of participants. Hence, the collected biological samples, including faeces, urine, HM, buccal swap, and umbilical cord blood samples, were obtained by non-invasive procedures that had been tested and employed in earlier studies carried out at HULAFE. SOPs for sample collection and handling were designed and reviewed by the study team ahead of time. Sample collection kits with detailed instructions and pictures were prepared for sample collection at home. Finally, all procedures were tested in a small pilot study conducted before the initiation of the main study, allowing for minor amendments in the protocols and leading to a homogeneous process conducted all through the main study. During follow-up, some visits were carried out at home avoiding the additional effort for participants to travel to the hospital. These efforts helped to encourage mothers to participate in the study and enhanced adherence during the follow-up period.

One of the most outstanding strengths of this study is the rich array of information obtained from participants during the first six months of life, a pivotal period for health programming. Direct access to participants through trained staff of the research team at the hospital greatly aided to reduce the rate of missing data. The promotion of good relationships with participants could facilitate to further extending the life of the study cohort. Nevertheless, the follow-up period presented challenges. We would like to highlight the loss of some samples due to technical issues when collected at home as well as the temporary loss of some sampling time points, e.g., due to illness of participants or overburdening of lactating mothers. Our preliminary results need to be confirmed in a greater cohort of infants and in different European settings.

This study will provide a better understanding of the impact of maternal nutrition in HM composition, and the interplay of HM composition, microbiota, and newborn physiology, especially in PI's development. More knowledge and a better understanding could allow for a personalized nutrition or even individualized care at an early stage of the neonatal period to improve the outcome of these vulnerable newborns. Additionally, data obtained from this study will be used to train a machine-learning algorithm for providing dietary advice to lactating mothers and will be implemented in a user-friendly platform based on a combination of user-provided information and biomarker analysis.

## 4. Conclusion

A better understanding of the factors affecting the milk's composition, together with the health implications for infants plays an important role in developing improved strategies of nutraceutical management in infant care. The development of a user-friendly platform based on a combination of user-provided information and biomarker analysis for providing dietary advice would make latest scientific advances directly accessible to a wide range of lactating mothers. This development is anticipated to be of special importance for health, growth, and development of prematurely born infants.
